# Recent advances in inhibition of porcine reproductive and respiratory syndrome virus through targeting CD163

**DOI:** 10.3389/fmicb.2022.1006464

**Published:** 2022-09-16

**Authors:** Xiaoxiao Zhang, Chunhe Guo

**Affiliations:** ^1^Key Laboratory of Zoonosis Prevention and Control of Guangdong Province, College of Veterinary Medicine, South China Agricultural University, Guangzhou, Guangdong, China; ^2^Guangdong Laboratory for Lingnan Modern Agriculture, Guangzhou, Guangdong, China

**Keywords:** PRRSV, CD163, gene-editing, targeting, receptor

## Abstract

Porcine reproductive and respiratory syndrome virus (PRRSV) has plagued the pig industry for more than 30 years and causes great economic losses. At present different commercial vaccines are available but limited tools. Until now at least six potential host factors are identified as the key receptors for PRRSV infection. Among them, CD163 molecule is the most important and critical in PRRSV life cycle responsible for mediating virus uncoating and genome release. It determines the susceptibility of target cells to the virus. Several PRRSV non-permissive cells (such as PK-15, 3D4/21, and BHK-21) are demonstrated to become completely susceptible to PRRSV infection in the presence of expression of porcine CD163 protein. Therefore, CD163 has become the target for the design of novel antiviral molecules disrupting the interaction between CD163 and viral glycoproteins, or the breeding of gene-modified animals against PRRSV infection. In this review, we comprehensively summarize the recent progress in inhibition of PRRSV replication *via* targeting CD163 receptor. In addition, whether there are other potential molecules interacting with CD163 in the process of uncoating of virus life cycle is also discussed.

## Introduction

Porcine reproductive and respiratory syndrome (PRRS), first reported in North America in 1987, is a highly contagious disease causing huge direct and indirect economic losses to the pig industry worldwide (Wang et al., 2021). It is characterized by heavy late-term reproductive failures in sows and respiratory disorders, anorexia and growth reduction in pigs of all ages (Amadori et al., 2021). The causative agent, PRRS virus (PRRSV), is a small, enveloped, single-stranded positive-sense RNA virus in the genus Betaarterivirus, family *Arteriviridae*. Its genome is about 15 kb in length with a 5′ cap and a 3′ poly (A) tail and includes at least 11 open reading frames (ORFs) expressed from genomic and subgenomic mRNAs (Ma et al., 2021). The first two ORFs (ORF1a and ORF1b) encompass about 75% of the genome and encode two large polyproteins (pp1a and pp1ab), which are proteolytically processed into approximately 14 viral non-structural proteins (nsps): nsp1α, nsp1β, nsp2–6, nsp7α, nsp7β, and nsp8–12 (Yu et al., 2021). Based on sequence analysis, PRRSV is divided into PRRSV 1 (species Betaarterivirus suid 1) and PRRSV 2 (species Betaarterivirus suid 2) by the International Committee on Taxonomy of Viruses (Amadori et al., 2021), which share about 70% nucleotide sequence identity (Zhang and Feng, 2021).

Porcine reproductive and respiratory syndrome virus has a very narrow host cell tropism (Welch and Calvert, 2010). The primary targets for the virus are cells of monocyte/macrophage lineage such as porcine alveolar macrophage (PAM). Additionally, African green monkey kidney cell line MA-104 and Marc-145 (a derivative of MA-104) support PRRSV infection and proliferation and are commonly used for virus study *in vitro* (Su et al., 2021). Several specific cellular factors were identified as entry mediators and receptors for the virus including heparan sulphate, CD163, CD169, CD151, non-muscle myosin heavy chain, and vimentin. Among them, CD163 is the most specific and indispensable receptor for virus uncoating and genome release both *in vivo* and *in vitro*. CD163 molecule is a 130 kDa macrophage-specific glycoprotein which is a member of scavenger receptor cysteine-rich superfamily (SRCR) (Islam et al., 2020). It consists of nine extracellular SRCR domains (SRCR1-9), a single transmembrane segment and one intracellular domain. Functionally, CD163 is well-described as a receptor for binding of tumor necrosis factor (TNF)-like weak inducer of apoptosis, pathogen entry into host cells, and hemoglobin clearance through hemoglobin-haptoglobin complexes, which is crucial for physical health. It also can interact with erythroblastic cells to improve the survival and growth of the cells and mediate anti-inflammatory responses (Van Gorp et al., 2010; Siwan et al., 2022). Several PRRSV non-susceptible cells such as PK-15, BHK-21, and 3D4/21 transfected with a plasmid expressing CD163 cDNA gain the ability to support PRRSV infection and proliferation (Whitworth and Prather, 2017). Marc-145 cells, susceptible for PRRSV 1 and 2 replication, express CD163 molecule but not CD169 (Zhang and Yoo, 2015), indicating that CD163 is a major and an essential receptor for PRRSV entry into target cells.

The objective of this review is to introduce the current progress in inhibition of PRRSV infection by targeting CD163 receptor and to explore the key amino acids in SRCR5 domain of CD163 receptor for editing CD163 molecule more precisely. Furthermore, the review also discusses whether there are other potential host cofactors that interact with CD163 to mediate virus uncoating and genome release, which lays the foundation for the development of new antiviral targets against PRRSV infection.

## Targeting CD163 mRNA

**FIGURE 1:**
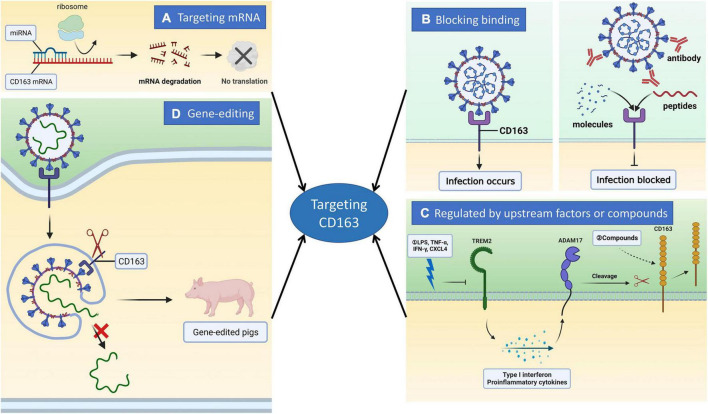
Inhibition of PRRSV infection by targeting CD163 molecule through different strategies. CD163 is a major receptor for PRRSV infection of cells. It is very promising to prevent and control PRRSV *via* targeting CD163. Commonly there are four approaches to regulate CD163 expression. **(A)** miRNAs are capable of binding to CD163 mRNA to induce its degradation, ultimately blocking the translation of CD163 protein. **(B)** CD163 interacts with viral glycoproteins such as GP2, GP3, and GP4 to mediate virus uncoating and genome release. Based on it, small molecules, peptides and antibodies can disrupt the interaction between CD163 and virus glycoproteins to block uncoating thus suppressing PRRSV proliferation. **(C)** CD163 is involved in anti-inflammatory responses and several factors are reported to has the ability to regulate its expression. Upstream factors such as TREM2 and ADAM17 can regulate CD163 expression to modulate PRRSV infection. TREM2 silencing causes the activation of PI3K/NF-κB pathway to boost the expression of pro-inflammatory cytokines. Therefore, the cleavage of CD163 molecule mediated by ADAM17 is occurred, ultimately restraining virus replication. Several compounds are also capable of modulating CD163 expression. **(D)** CD163 is an essential receptor for PRRSV. CRISPR/Cas9-mediated gene modification enables us to edit CD163 to construct genetically modified pigs. The reason why the animals show completely resistance to PRRSV infection is that the modified CD163 cannot bind to the virus glycoproteins thereby blocking uncoating.

MicroRNAs (miRNAs), small non-coding RNAs and 19–25 nucleotides in size, are considered to be highly conserved among different species which regulate gene-expression post-transcriptionally or inhibit their translation (Correia de Sousa et al., 2019; Hill and Tran, 2021; Figure 1A). The sequences of mature miRNAs which are guided to the end of their target mRNAs *via* base pairing are located in exons of non-coding RNAs or introns. It is no doubt that miRNAs play important roles in regulation of physiological process such as cell proliferation, differentiation, development and apoptosis as well as host-pathogen interactions, embryogenesis, metabolism and tumorigenesis (Cai et al., 2009; Saliminejad et al., 2019). miRNAs are isolated from plasma, serum, tissues, cells and so on (Lu and Rothenberg, 2018). Long non-coding RNAs (lncRNAs), defined as RNA transcripts of more than 200 nucleotides in length that do not encode protein owing to the lack of open reading frame, have also shown to be involved in many cellular processes including transcription, translation, metabolism, differentiation and occurrence of diseases (Kopp and Mendell, 2018; Ali and Grote, 2020; Bridges et al., 2021). Commonly the expression of lncRNAs is correlated with the expression of nearby genes, and they interact with RNA, DNA, protein or a combination of them to regulate chromatin dynamics, protein assembly, and transcription (Xing et al., 2021). Recent studies have reported that lncRNAs play crucial roles in viral infection.

Blood monocytes are less susceptible to PRRSV than PAMs, and the expression level of ssc-miR-181c is much higher than that in PAMs, indicating that ssc-miR-181c may be capable of targeting CD163 mRNA to downregulate its expression thus suppressing PRRSV infection. As expected, ssc-miR-181c decreases the translation level of CD163 in both PAMs and blood monocytes *via* binding the 3′ untranslated region of CD163 mRNA (Gao et al., 2013). Using an RNA-induced silencing complex immunoprecipitation assay, ssc-miR-181c binds to CD163 mRNA to inhibit PRRSV entry into PAMs and it has the potential to develop into therapeutics against PRRSV infection. Furthermore, PRRSV infection leads to the decrease of ssc-miR-124a expression which could mediate the downregulation of CD163 mRNA and protein levels by targeting the CD163 mRNA sequence (Li et al., 2021). Similarly, the expression of CD163 receptor both at mRNA and protein levels were potently suppressed by adenovirus- or exosome-delivered artificial miRNAs, thus causing the inhibition of PRRSV replication (Zhu et al., 2014). Gao et al. (2021) reported that CD163-lncRNAs correlation network was constructed and found that the expression of 38 lncRNAs that are correlated with CD163 are modulated by PRRSV infection. lncRNAs may elevate the expression of CD163 to promote virus replication in cells, suggesting that lncRNAs may be involved in PRRSV entry through modulating CD163 expression. Therefore, miRNAs and lncRNAs can target CD163 mRNA to affect PRRSV replication.

## Blocking porcine reproductive and respiratory syndrome virus binding to CD163 *via* interfering with the interaction between CD163 and viral glycoproteins

Viral structural proteins (glycoproteins) are the first and foremost step to interact with host factors on the cell membrane such as receptors in virus life cycle. Therefore, it is promising to develop molecular drugs targeting virus glycoproteins or host receptors. Previous studies have shown that PRRSV glycoproteins such as GP2, GP3, GP4, and GP5 interact with host receptor CD163 to mediate virus entry and uncoating (Stoian et al., 2022). There are various strategies to block virus uncoating. CD163 receptor represents a key target for designing small molecules to block uncoating (Figure 1B). One approach that blocking the interaction of viral glycoproteins with CD163 is by antibodies (Figure 1B). Xu H. et al. (2020) reported that monoclonal antibodies (mAbs) 6E8 and 9A10 against scavenger receptor cysteine-rich domain (SRCR) 5–9 of CD163 molecule were produced and characterized which can inhibit various PRRSV strains in both PAMs and Marc-145 cells either prior to or after attachment. Mechanistically, treatment with either mAb causes a dramatic reduction of CD163 mRNA expression in PAMs and Marc-145 cells, which contributes to the inhibition of PRRSV replication. Consistently, a monoclonal antibody against SRCR5-6 region of CD163 molecule was also shown to partially suppress PRRSV infection in PAMs, and this inhibition is a dose-dependent manner (Zhang et al., 2020b). In addition, the recombinant SRCR5-9 protein from porcine CD163 was purified and produced, which could bind to different PRRSV strains thus displaying a potent anti-PRRSV activity (Han et al., 2022). To test the role of the soluble virus receptor in PRRSV infection, soluble CD163 protein was expressed and purified using adenoviral expression system and is capable of binding to virus particles to block virus infection of PAMs (Chen et al., 2014). In addition, its anti-PRRSV property was evaluated in piglets. The soluble CD163 protein was successfully expressed *in vivo* using ELISA and pigs inoculated with adenoviral vector expressing soluble CD163 were protected from infection with highly pathogenic PRRSV strains as showing alleviated viremia, viral loads, and clinical symptoms (Xia et al., 2018). Therefore, the soluble CD163 protein has the potential to develop into a novel antiviral against PRRSV. CD163 has also been explored as a target for the development of small molecules (Figure 1B). The mimics of receptor protein could strongly block CD163 binding to viral glycoproteins. Using a cell-based bimolecular fluorescence complementation (BiFC) assay, a list of small molecules targeting CD163 SRCR5 domain were screened. Among these compounds, 4-Fluoro-2-methyl-N-[3-(3-morpholin-4-ylsulfonylanilino)quinoxalin-2 yl]benzenesulfonamide shows an inhibitory effect on the interaction between CD163 molecule and viral glycoproteins, resulting in the suppression of both type I and II PRRSV strains (Huang et al., 2020a). Collectively, the interference targeting viral glycoproteins-CD163 interaction would be a novel strategy for PRRSV control and eradication.

## Regulation of CD163 expression by upstream factors or compounds

CD163 molecule, a specific marker of monocytes and macrophages, is involved in anti-inflammatory responses. It is also a therapeutic target due to its association with inflammatory process. The expression of CD163 is tightly modulated in a tissue or differentiation-specific manner. A number of factors have been reported to regulate CD163 expression (Etzerodt and Moestrup, 2013; Figure 1C). The potent stimulators that could upregulate CD163 expression include glucocorticoid, IL-6, IL-10, hemoglobin, and macrophage colony stimulating factor, while the factors downregulating its expression are interleukin-4 (IL-4), IL-13, IL-1α/β, IFN-γ, lipopolysaccharide (LPS), TNF-α, transforming growth factor-beta, granulocyte macrophage colony stimulating factor, hypoxia, and cross-linking of Fc gamma receptor (Etzerodt and Moestrup, 2013). Similarly, 12-O-tetradecanoylphorbol-13-acetate (TPA) treatment leads to reduced expression of CD163 thus hindering virus infection (Patton et al., 2009). Some chemokines such as CXC-chemokine ligand 4, CXC-chemokine ligand 8 and macrophage inflammatory protein-1 alpha are also involved in regulation of CD163 expression (Kowal et al., 2011). Furthermore, activation of the cell membrane toll-like receptors (TLRs) including TLR-2, –4, and –5 results in the upregulation and activation of the disintegrin and metalloproteinase 17 (ADAM17), leading to ectodomain shedding and downregulation of CD163 on cell membrane (Kowal et al., 2011; Gomez-Laguna et al., 2013; Lisi et al., 2014; Figure 1C). Mechanistically, LPS, a specific agonist to TLR4, activates TLR4-NF-κB pathway to induce the expression of proinflammatory cytokines in PAMs. Therefore, CD163 is cleaved by ADAM17 activation, which results in the limitation of PRRSV replication (Zhu et al., 2020a). Previous literature also demonstrated that ADAM17 suppression elevates the expression of CD163 receptor to upregulate PRRSV entry into Marc-145 cells and PAMs, while overexpression contributes to its inhibition of CD163 and PRRSV replication (Guo et al., 2014). In addition, proprotein convertase subtilisin/kexin type 9 (PCSK9) exhibits a strong anti-PRRSV effect which is attributed to the degradation of CD163 receptor mediated by it, and the antiviral activity could be antagonized by viral non-structural protein 11, indicating that PCSK9 has the ability to modulate CD163 expression (Zhang et al., 2020a). Our recent study revealed that triggering receptor expressed on myeloid cells 2 (TREM2), an anti-inflammatory factor, is closely associated with PRRSV infection and proliferation (Zhu et al., 2020b). TREM2 silencing causes the activation of PI3K/NF-κB pathway to boost the expression of pro-inflammatory cytokines. As a result, the cleavage of CD163 molecule mediated by metalloproteinase ADAM17 is occurred, ultimately restraining virus proliferation (Figure 1C). Overall, TREM2 regulates CD163 expression through PI3K/NF-κB pathway and inflammatory responses. In addition to CD163, there are other mediators involved in PRRSV entry into target cells. Non-muscle myosin heavy chain 9 (MYH9), identified as a cell receptor for PRRSV, interacts with CD163 to promote virus infection (Hou et al., 2019). Further study showed that the effect is attributed to the interaction of the ectodomain SRCR1-4 of CD163 with the C-terminal domain of MYH9 without involvement of other factors. This report suggests that the role of CD163 in mediating PRRSV internalization requires its partner MYH9. In addition to upstream factors, several compounds can also modulate CD163 expression. Dexamethasone, a potent steroidal anti-inflammatory drug, has the ability to improve the expression of CD163 in immortalized porcine kidney-derived macrophages (IPKM) (Takenouchi et al., 2021). This effect induced by dexamethasone can be blocked by the inhibitor of p38 mitogen-activated protein kinase (MAPK), indicating that p38 MAPK pathway takes part in regulation of dexamethasone-induced CD163 expression. Cryptotanshinone, a natural compound, is capable of limiting the activation of signal transducer and activator of transcription 3 and the expression of IL-10 to inhibit CD163 expression, ultimately leading to suppression of replication of different PRRSV strains (Huang et al., 2020b). Taken together, the level of CD163 expression could be regulated by its upstream host factors or exogenous compounds, which hold great promise for the research of anti-PRRSV strategies.

## Targeting CD163 *via* gene-editing

Clustered Regularly Interspaced Short Palindromic Repeats (CRISPR)/CRISPR-associated protein 9 (Cas9) technology is widely acknowledged for having a reliable, efficient, and available tool for gene editing, epigenetic modulation, and transcriptional perturbation (Barman et al., 2020; Figure 1D). CRISPR/Cas9 system-mediated genome modification enables us to treat diseases including infections, cancer, genetic disorders, neurodegeneration, cystic fibrosis, and immunological disorders (Doudna and Charpentier, 2014; Jiang and Doudna, 2017). The first gene-edited pigs lacking functional CD163 receptor were produced by Whitworth et al. (2014) from University of Missouri-Columbia, USA. The CD163 knockout pigs were generated using CRISPR/Cas9 and somatic cell nuclear transfer technologies. The animals confer resistance to PRRSV infection as showing no clinical symptoms, viremia, and antibody responses (Whitworth et al., 2016). Duroc pigs with the deletion of CD163 gene are also not susceptible to infection by highly pathogenic PRRSV stain TP (Yang et al., 2018), indicating that PRRSV infection of different breeds of pigs is dependent on membrane CD163 receptor. Since CD163 molecule has important biological functions including participating in the initiation and perpetuation of the inflammatory responses and mediating the removal of hemoglobin from blood plasma upon intravascular hemolysis, and soluble CD163 is capable of limiting T cell proliferation in a dose-dependent manner (Hogger and Sorg, 2001; Frings et al., 2002), the complete knockout of it may have key physiological effects on animal development and growth as well as the susceptibility to infection with other pathogens. Therefore, gene-edited pigs with the deletion of SRCR5 domain of CD163 were generated by CRISPR/Cas9 in zygotes. As expected, the animals are healthy and the expression and folding of CD163 gene of PAMs and peripheral blood monocytes (PBMCs) isolated from CD163 SRCR5-deleted pigs are comparable to that of wild type animals (Burkard et al., 2017). Further, PAMs display fully resistance to PRRSV 1 and 2 infections and pigs are not susceptible to infection with PRRSV 1 (Burkard et al., 2018). These literatures demonstrate that the SRCR5 domain of CD163 receptor mediates PRRSV uncoating in endosome thus promoting viral genome release into cytoplasm. Consistently, other study also showed that challenge of CD163 SRCR5-edited pigs with highly pathogenic PRRSV (HP-PRRSV) confers resistance to virus infection and the biological functions of CD163 are intact (Wang et al., 2019). Additionally, substitution of porcine CD163 exon 7 encoding SRCR5 with the corresponding region of human CD163-like 1 (hCD163L1) SRCR8 exerts a strong anti-PRRSV effect by suppressing uncoating and genome release *in vitro* and *in vivo* (Chen et al., 2019). Wells et al. (2017) also produced above CD163-modified pigs and found that the animals show resistance to PRRSV 1 but not 2, suggesting that PRRSV 1 and 2 highly differ in recognition of CD163 receptor. PRRSV infection is capable of crossing the placental barrier and infecting fetuses in dam thereby leading to abortion and fetal death. Recent literature demonstrated that the presence of CD163 knockout genotype of dam has the ability to protect fetuses from infection by PRRSV (Prather et al., 2017). Our previous research revealed that the modified Marc-145 cell, which is deleted of SRCR5 domain of CD163, is not susceptible to infection with various PRRSV strains, and calpain 1 is identified as a novel host factor that interacts with CD163 to facilitate virus uncoating using the gene-modified cell line (Yu et al., 2019). The ligand binding pocket of SRCR5 domain of CD163 may be involved in interaction with viral glycoproteins such as GP2a and GP4 to mediate uncoating. To preserve the functions of CD163 molecule as much as possible, we generated gene-modified pigs harboring a partial deletion of CD163 SRCR5 which contains ligand binding pocket (Guo et al., 2019). As expected, the animals are fully resistant to infection by PRRSV 2 strains as showing no clinical signs, pathological abnormalities or antibody responses. PAMs isolated from the pigs show a similar phenomenon. Like PRRSV, transmissible gastroenteritis virus (TGEV) is an enteric coronavirus associated with lethal severe diarrhea in pigs causing huge economic losses worldwide. Based on it, double gene knockout pigs lacking two receptor proteins CD163 and pAPN were generated using CRISPR/Cas9 approach and show full resistance to PRRSV 2 and TGEV (Xu K. et al., 2020). Moreover, the animals display reduced susceptibility to porcine delta coronavirus infection, suggesting that pAPN is a receptor mediating porcine delta coronavirus entry into target cells. There are no differences in meat production performance and reproductive performance between double gene knockout and wild type pigs.

## Concluding remarks

Porcine reproductive and respiratory syndrome continues to affect pig industry worldwide. Various commercial vaccines are available and valuable. However, they provide only limited protection and have no cross-protection against heterologous strains, they also have other drawbacks such as long viremia and serious side effects. Therefore, vaccination is highly controversial. Since CD163 is an indispensable factor for PRRSV infection, we focus on the recent advances in inhibition of PRRSV *via* targeting CD163 in this review. Commonly, there are four strategies regulating or blocking its expression (Figure 1): (I) targeting CD163 mRNA by miRNAs or lncRNAs; (II) blocking PRRSV virions binding to CD163 *via* interfering with the interaction of CD163 with viral glycoproteins; (III) modulation by upstream host factors or exogenous compounds; (Iv) gene-editing. miRNAs and lncRNAs are easily degraded, thus limiting their application. Gene-editing is the most promising for breeding CD163-modified pigs against PRRSV infection in pig farms.

**FIGURE 2:**
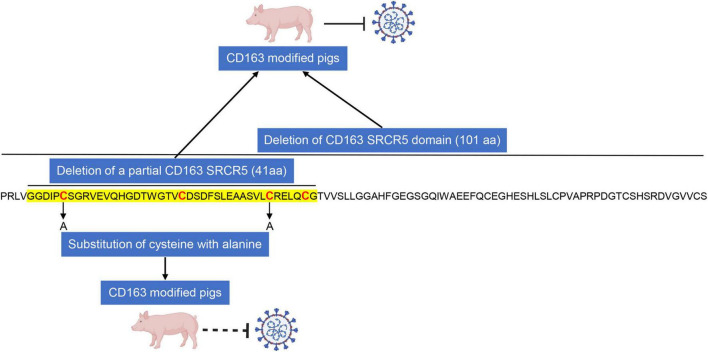
CD163 modified pigs may not be susceptible to infection with PRRSV by performing cysteine to alanine replacements in SRCR5 domain. Deletion of entire or partial CD163 SRCR5 domain confers resistance of pigs to PRRSV infection. Furthermore, substitution of cysteine at positions 10 and 39 with alanine in SRCR5 region of CD163 can render cells resistant to infection by PRRSV, indicating that cysteine residues in SRCR5 domain of CD163 play critical roles in mediating virus uncoating and replacement of cysteine with alanine in SRCR5 would be enough to confer resistance of CD163 modified pigs to PRRSV. The location of deletion of 41 amino acids in our previous study (Guo et al., 2019) are indicated in sequence of SRCR5 region and the cysteine location are labeled in red.

**FIGURE 3:**
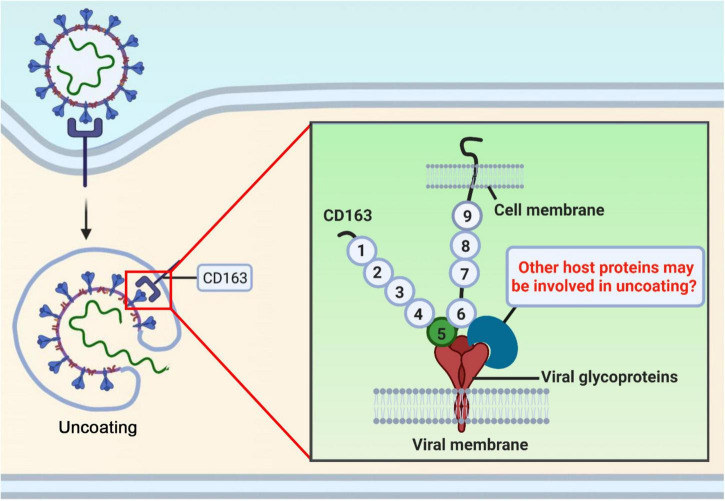
There may be other potential host cofactors that interact with CD163 to mediate virus uncoating and genome release. CD163 receptor determines whether virions uncoating is occurred in PRRSV life cycle. However, the molecular mechanism of uncoating remains largely unclear. Other molecules may participate in this process, which could be used as targets for gene-editing to render pigs resistant to PRRSV.

CRISPR/Cas9 gene-editing technology has been applied in animal field to breed disease-resistant pigs (Figure 1D). Pigs with a deletion of either full-length CD163 or the entire SRCR5 domain of CD163 or the partial SRCR5 domain including ligand binding pocket exhibit complete resistance to infection with PRRSV, indicating that CD163 SRCR5 domain is the binding site of the virus. However, the exact amino acid sequences within SRCR5 domain involved in PRRSV infection remain unclear. To address it, mutational research within CD163 SRCR5 was performed and all four disulfide bonds in SRCR5 are critical and required to support PRRSV infection and proliferation (Stoian et al., 2022). Further, replacement of cysteine at positions 10, 39, 70, and 90 with alanine in SRCR5 region confers resistance of cells to PRRSV infection (Stoian et al., 2022), which suggests that these amino acids are necessary for virus infection (Figure 2). This research also provides an explanation for our study that pigs lacking 41 amino acids which includes three disulfide bonds within SRCR5 domain are not susceptible to infection by PRRSV. What is more important, these data provide clues for modifying CD163 molecule more accurately to construct gene-edited pigs against PRRSV infection in the future (Figure 2). CD163 is a scavenger receptor playing an important role in clearance of hemoglobin and pathogens in host innate immune responses. It determines whether virions uncoating is occurred. However, the molecular mechanism of uncoating process remains largely unclear. In addition to CD163, we need to seek other targets for preventing and controlling PRRSV. There may be other potential host cofactors that interact with CD163 to mediate virus uncoating and genome release (Figure 3), which could be used as targets for gene-editing to render pigs resistant to PRRSV infection.

## Author contributions

CG: conceptualization, writing—review and editing, visualization, project administration, and funding acquisition. XZ and CG: writing—original draft preparation. Both authors read and agreed to the published version of the manuscript.
